# The color stability of artificial white spot lesions treated with resin infiltration after exposure to staining beverages

**DOI:** 10.1186/s12903-024-04693-w

**Published:** 2024-08-14

**Authors:** Apinya Limvisitsakul, Anisha Komalsingsakul, Pongsakorn Thamsrithip, Yod Thammasupapong, Supada Chaisomsukrudee, Sorawit Aramlerttrakul, Pisol Senawongse

**Affiliations:** 1 Division of Operative Dentistry, Department of Operative Dentistry and Endodontics, Faculty of Dentistry, Mahidol University, 6 Yothi Road, Ratchathewi District, Bangkok, 10400 Thailand; 2 Faculty of Dentistry, Mahidol University, 6 Yothi Road, Ratchathewi District, Bangkok, 10400 Thailand

**Keywords:** Color stability, Resin infiltration, White spot lesions, Staining

## Abstract

**Background:**

To evaluate the effect of staining beverages on the color-changing of resin-infiltrated artificial white spot lesions (WSLs).

**Methods:**

Thirty-five artificial WSLs were created by pH cycling on flat bovine teeth specimens. The WSLs were treated with resin infiltration and were divided into five groups based on staining beverages: artificial saliva, coffee, wine, green tea, and Coca-Cola. These specimens were subjected to a 28-day exposure to the respective beverages. Color stability was assessed using a spectrophotometer at different time points: baseline, after 7, 14, 21, and 28 days of exposure, and repolishing. The color difference (∆E) between each time point and the baseline was calculated. Statistical analysis was performed using two-way measures ANOVA with a significance level of *p* = 0.05.

**Results:**

All resin-infiltrated specimens exposed to staining beverages for 7 days exhibited more significant color changes than those exposed to artificial saliva. The color change patterns varied based on the type of beverage. The color alterations intensified with extended immersion in the wine and Coca-Cola groups, while there were no significant differences in the color of specimens after 28 days of immersion in the coffee and green tea groups. However, after cleaning with pumice powder, all specimens showed significantly reduced color changes compared to those observed after 28 days of immersion, except those immersed in coffee.

**Conclusions:**

Exposure of resin-infiltrated bovine tooth specimens to staining beverages resulted in a significant color alteration as the immersion time increased. However, the staining effect could be minimized by cleaning with pumice powder, except for the coffee group.

**Clinical relevance:**

After resin infiltration treatment, patients should be advised to minimize the consumption of colored beverages to prevent staining that could impact esthetic appearance.

## Background

Initial enamel caries or white spot lesions (WSLs) are characterized by a pseudo-intact surface layer and subsurface porosity resulting from enamel demineralization [1]. These porous lesions display a white chalky opaque appearance, causing patients aesthetic concerns. Various treatments for white spot lesions in the esthetic area are available, including remineralization, microabrasion, veneering, and the recently introduced resin infiltration technique [2].

With a minimally invasive approach, remineralization is a noninvasive first-line treatment option to preserve tooth structure whenever possible [3]. Remineralization involves applying a remineralizing agent and allowing the tooth to undergo natural remineralization over time [4]. However, this method requires long-term treatment and cannot be completed in a single visit.

On the contrary, microabrasion is a relatively invasive technique that involves using acid and an abrasive compound to remove the most superficial layer of defective enamel. Although it can be completed in a single visit, it does result in the loss of some superficial enamel layer [5]. Microabrasion may not be sufficient in more complex situations, such as extensive and deep enamel defects or multiple enamel defects with malalignment. When the previously mentioned treatments (remineralization and microabrasion) fail to achieve the desired results, veneering treatment is often recommended. Veneering offers excellent esthetic outcomes, but it is also the most invasive option available [6].

In 2010, a new technique called resin infiltration was introduced under the name Icon^®^ (DMG America Company, Englewood, NJ), which is known as a micro-invasive technology. The principle of the technology is to occlude the enamel porosities with resin by capillary action. This method entails etching the tooth surface with acid and filling the lesion porosities with a low-viscosity resin-based material. Using a resin with a refractive index close to hydroxyapatite achieves refractive index conformity between the lesion area and sound enamel [7]. As per the manufacturer’s instruction for resin infiltration, the 15% hydrochloric acid (Icon Etch) is applied to the lesion site for 2 min. After etching, the lesion area is rinsed with water for 30 s and dried with oil and water-free air. Next, 99% ethanol (Icon Dry) is applied to the lesion for 30 s. The whitish-opaque lesion should fade when the lesion is wetted with Icon Dry. If the whitish-opaque lesion persists, the etching process with Icon Etch may be repeated up to two times. After visually confirming the lesion’s appearance, it is dried with air to remove water. Then, the resin infiltrant (Icon) is applied over the lesion and left for 3 min to penetrate the microporosities of the lesion. Excess material is removed by a cotton roll and dental floss. The infiltrant is polymerized for 40 s using a light curing unit. Finally, the surface is polished using a polishing cup. Resin infiltration is considered a micro-invasive procedure as it only involves minimal loss of tooth structure [8]. Despite its advantages, resin-based materials are prone to color alteration over time. The primary cause of resin discoloration is exposure to external factors such as colored foods, beverages, and smoking [9–12]. This raises the question of whether applying resin-based material to enamel porosities makes them more susceptible to staining than normal enamel. Resin infiltration application is a relatively new technique; the purpose of this study was to evaluate the color stability of resin infiltration by determining color with the CIELAB system using a spectrophotometer on the flat enamel area of bovine tooth windows after immersing them in different staining solutions. The null hypothesis of this study was the color change of the demineralized enamel surface treated with resin infiltration would remain unchanged after exposure to either staining beverages for up to 28 days or the repolishing process conducted after staining.

## Methods

Thirty-five extracted bovine teeth, stored in chloramine-T solution at room temperature, were used in this study within one month after extraction. The protocol of this study was approved by the Mahidol University-Institute Animal Care and Use Committee (MU-IACUC) with the protocol number F02-63-007. Before experimentation, the teeth were carefully examined under a stereomicroscope (Eclipse E400 POL, Nikon, Tokyo, Japan) to ensure the absence of cracks or other surface defects and kept in tap water. The buccal surface of each extracted tooth was ground flat with 320, 600, and 1200 grit of silicon carbide papers (Carbimet, Buehler, Lake Bluff, IL, USA) under water and then wet-polished with felt paper using diamond paste (1 μm MetaDi Diamond Pastes, Buehler) to expose a flat enamel surface. A rectangular window of size 5 × 5 mm was prepared on each specimen’s enamel surface by covering the surrounding surfaces with nail varnish (Nail Enamel, Revlon, New York, NY). The artificial initial caries were created on the prepared enamel surface with a pH cycling technique for ten days, according to Ten Cate and Duijsters [13]. First, all specimens were immersed in demineralization solution 1 (pH 4.4) (comprising 2.2mM CaCl_2_, 2.2mM NaH_2_PO_4_.2H_2_O, and 0.05 M acetic acid) for 3 h. Next, the specimens were immersed in remineralization solution (pH 7.0) (1.5mM CaCl_2_, 0.9mM NaH_2_PO_4_.2H_2_O, and 0.15 M KCl) for 2 h. Then, all specimens were immersed in demineralization solution 2 (pH 4.7) (containing 2.2mM CaCl_2_, 2.2mM NaH_2_PO^4^.2H_2_O, and 0.05 M acetic acid) for 3 h. Finally, the specimens were again immersed in a remineralization solution for 16 h. The initial baseline color of all the specimens was assessed using the vita shade mode of a spectrophotometer (SpectroShade Micro II, Spectroshade USA).

Following the manufacturer’s instructions, all demineralized specimens were treated with resin infiltration using Icon^®^ (DMG America Company, Englewood, NJ, USA). First, the Icon Etch was applied to the artificial white spot lesion. The etching agent was left on the surface for 2 minutes. After the appropriate etching time, thoroughly rinse off the etching agent with water for 30 s was applied. The etched enamel surface was completely dried. Next, the Icon Dry was applied to the lesion for 30 s, and then the lesion was dried with air to remove water. The low-viscosity infiltrant resin was applied and left for 3 min according to the manufacturer’s instructions. After applying the resin, 40 s of light-curing was employed to polymerize the resin with a light curing unit (Bluephase ^®^ 20i, Ivoclar Vivadent, Schaan, Liechtenstein) with an intensity of about 1,100 mW/cm^2^. Finally, the specimens were polished with a prophy cup and pumice to achieve a smooth surface. Each specimen’s initial L*, a*, and b* values (using the Commission Internationale de l’Eclariage Lab* (CIELAB) system) were assessed using a spectrophotometer. This color-determining process was performed in a dark box. The device’s tip was held perpendicular to the tested enamel window when performing the test. The CIELAB measurement performed after completing the resin infiltration procedure was recorded as C0.

The resin-infiltrated specimens were divided into five groups of seven specimens (*n* = 7 per group) for immersion in one of the following solutions: (1) artificial saliva, (2) black coffee (Nescafe Instant Coffee Americano, Nestle (Thailand), Bangkok, Thailand), (3) red wine (George Wyndham, Wyndham Estate, New South Wales, Australia.), (4) Coca-Cola^®^ (Coca Cola Company(Thailand), Bangkok, Thailand), and (5) green tea (Ito En company, Tokyo, Japan). To prepare the black coffee solution, two grams of coffee were mixed with 140 ml of water, filtered, and poured into the containers according to the manufacturer’s instructions [14, 15]. Each specimen was immersed in one vial containing 1 ml of each respective solution. For group 1, control, the specimens were kept in artificial saliva (KCl 2236.5 mg, KH_2_PO_4_544.4 mg, CaCl_2_.2H_2_O 77.7 mg, MgCl_2_19 mg, C_6_H_18_N_2_O_4_S 4766.2 mg in Deionized water 1000 ml) for 28 days. For groups 2 to 5, experimental groups, the specimens were immersed in the respective beverage for 10 min daily, followed by storage in artificial saliva for another 23 h and 50 min, and this process was repeated for 28 days. The staining solution was replaced with a fresh solution daily for 28 days. After 28 days of immersion, all specimens were polished with pumice powder using a prophy cup. Color assessment was conducted using CIELAB measurements on days 7, 14, 21, and 28 and after polishing (C7, C14, C21, C28, CP, respectively). The differences in color at any measured day (ΔE) were calculated by comparing them with the color on the day0 using the following specific formula:

$$\eqalign{& \Delta E = [{\left( {{L_{after}}* - {L_{before}}*} \right)^2} + {\left( {{a_{after}}* - {a_{before}}*} \right)^2} \cr & \,\,\,\,\,\,\,\,\,\,\,\,\, + {\left( {{b_{after}}* - {b_{before}}*} \right)^2}{]^{1/2}} \cr}$$

**Fig. 1:**
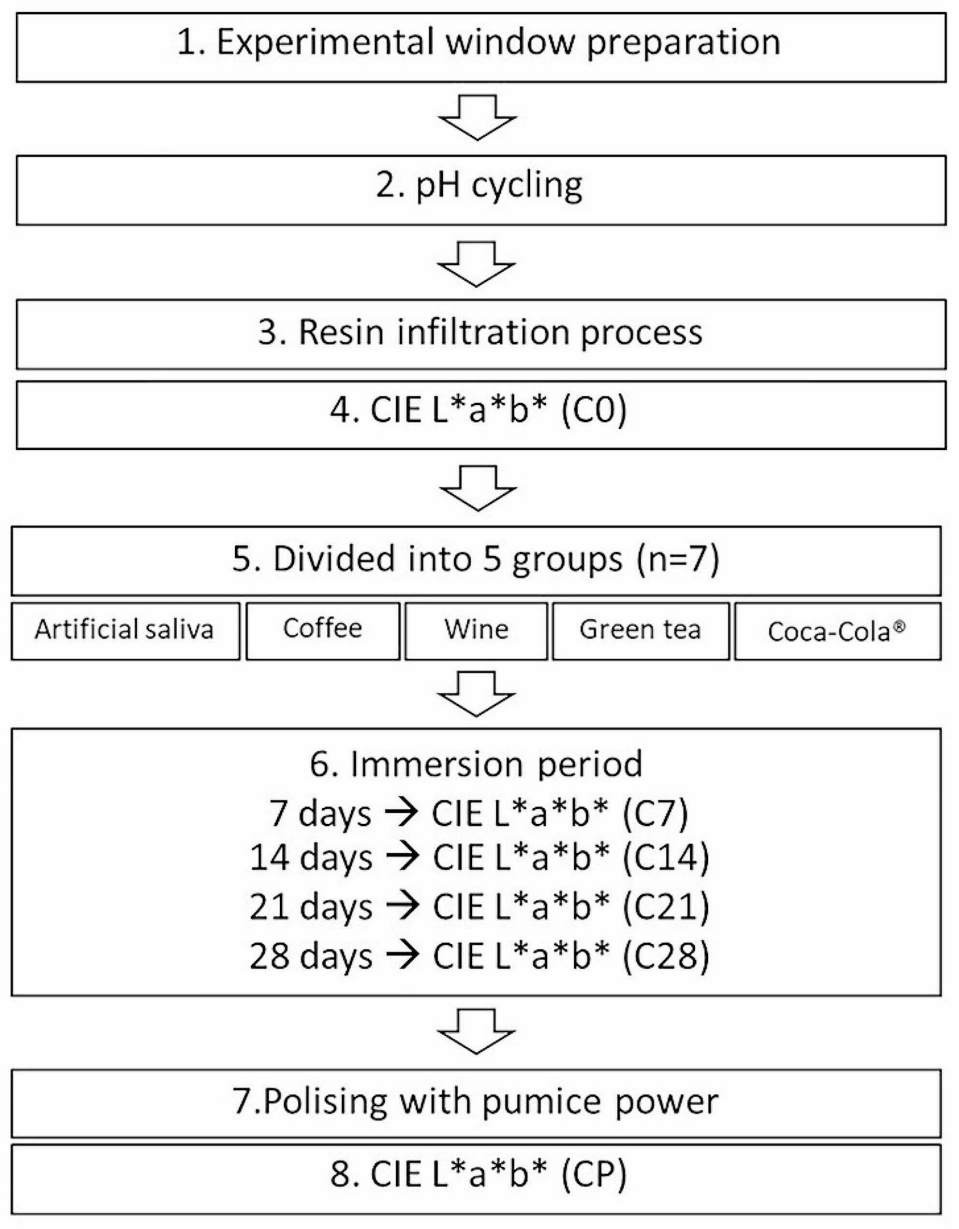
Process for specimen preparation

All specimen preparation processes are demonstrated in Fig. 1.

Statistical analysis was performed with IBM SPSS statistics version 26 (IBM, New York, United States). The normal distribution and homogeneity of variances of ΔE were verified using the Kolmogorov–Smirnov and Levene’s tests. Subsequently, a two-way measures ANOVA was conducted to analyze the influence of the intervention methods and the type of staining solutions. A Dunnett post hoc test (*p*-value = 0.05) was used for multiple comparisons.

## Results

**Fig. 2:**
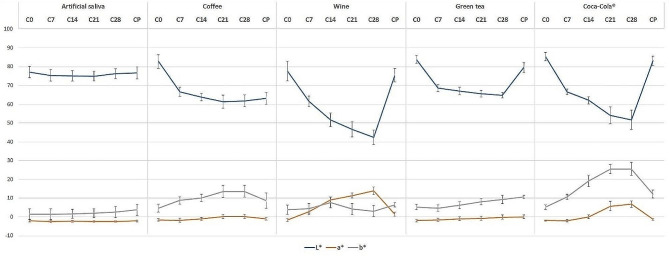
Means and standard deviations of the L* a* b* value after immersion in artificial saliva or staining beverages and repolishing

The L*a*b* values are shown in Fig. 2. The L* value decreased in all samples after beverage immersion, indicating a reduction in brightness. The most substantial decreases were observed in the wine and Coca-Cola^®^ groups. On the other hand, the a* value increased in all samples except the artificial saliva group, with the most significant increase found in the wine group. Similarly, the b* value increased in all groups. Therefore, the most notable increase was observed in the Coca-Cola^®^ group.

**Fig. 3:**
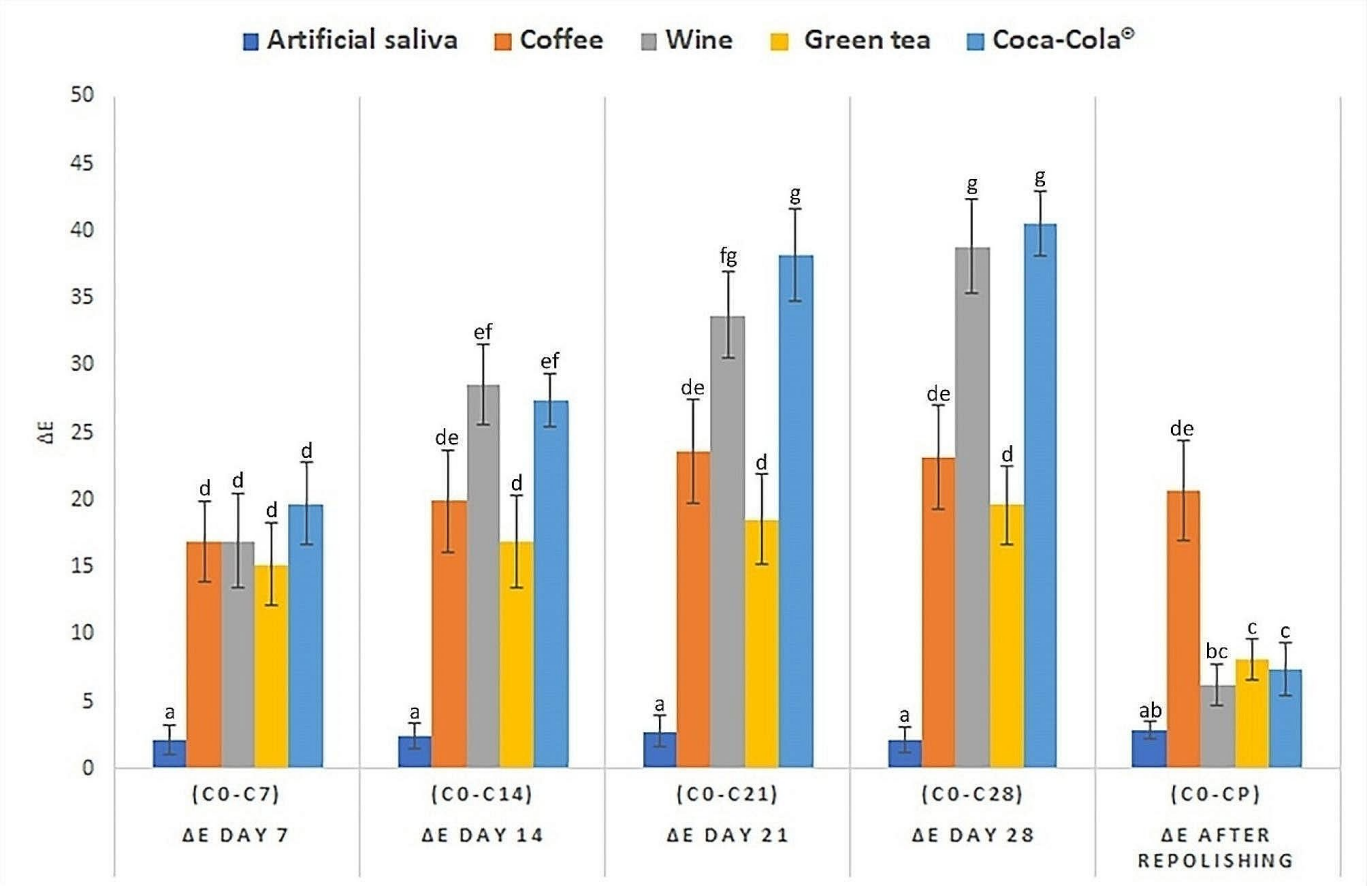
Means and standard deviations of the color difference (ΔE) comparing the initial color of the white spot lesion treated with resin infiltration with any observing period after immersion or repolishing. Differences in superscript alphabets indicate statistically significant differences

Figure 3 displays the color changes observed in specimens immersed in staining beverages for 28 days, with color measurements taken every 7 days. Additionally, the bar chart illustrates the color change values of specimens after being polished with pumice powder following the 28 days of immersion period. The results show noticeable color changes occurred in all groups after 7 days of beverage immersion, compared to the artificial saliva group, which served as the control group (*p* < 0.05). The pattern of color changes varied among the different beverages and periods, indicating the susceptibility of resin-infiltrated specimens to staining over time.

No statistically significant changes in ΔE were noted in the artificial saliva groups during the 28 days of immersion period and after repolishing. In the coffee group, although the color change values were significantly higher than those of the control group during the initial immersion (*p* = 0.000), no significant differences in color changes were observed throughout the 28 days of immersion. Additionally, there was no significant difference in color change values after polishing. For the green tea group, no significant differences in color changes were observed throughout the 28-day immersion period, similar to the coffee group. However, unlike the coffee group, a statistically significant difference in color change values was observed after polishing (*p* = 0.001).

In the wine and Coca-Cola groups, a progressive increase in color change was observed over the immersion period. This study’s most significant color changes were shown in the Coca-Cola group after 21 and 28 days of immersion and in the wine group after 28 days. However, both groups significantly reduced color change values after polishing (*p* = 0.000). Nevertheless, the results indicate that even after polishing, the specimens in the coffee, green tea, and Coca-Cola groups still exhibited significantly higher color change values than the artificial saliva group (*p* = 0.001,0.003,0.042, respectively).

## Discussion

Based on the results of this study, the null hypothesis was rejected. There were significant differences in color change after exposure to the staining beverages and repolishing after staining. The repolishing procedure significantly decreased the intensity of color changes caused by almost all staining beverages, except for coffee which had no significant effect on decreasing the degree of color changes between exposure to the coffee and repolishing after staining.

Several methods have been introduced for the evaluation of tooth colors including tooth color shade guides, colorimeters, and spectrophotometers. Visual shade matching is a method that involves visually comparing the tooth color to a standard shade guide and selecting the shade that closely matches the tooth color. Colorimeters are handheld devices that measure tooth color by illuminating the tooth surface with light and analyzing the reflected light. They provide tooth color measurements. Spectrophotometry measures tooth reflectance and absorption of light to quantify their color. This method provides quantitative color data [16]. The spectrophotometer used in this study presents the CIE-L*a*b* data. The CIE-Lab color sphere is a standardized color representation system divided into three dimensions (L*, a*, and b*). The brightness dimension is the L* component. The L* value ranges from 0 (pure black) to 100 (pure white). Higher L* values indicate brighter colors, while lower values indicate darker colors. The color intensity is represented by a*(green-red axis) and b*(yellow-blue axis). The a* component represents the position of a color along the green-red axis. Positive values indicate a reddish hue, while negative values indicate a greenish hue. The b* component represents the position of a color along the blue-yellow axis. Positive values indicate a yellowish hue, while negative values indicate a bluish hue. The color difference (ΔE) of two samples was calculated from the sample color alteration in all axes. Therefore, the ΔE parameter was used to indicate the color changes in this study.

Due to the spectrophotometer’s tooth color perception being associated with light reflection and absorption, a standardized flat enamel surface is necessary for the experimental measurement. Bovine incisors were used in this study because of their large size. Compared with human enamel, the chemical and physical properties of bovine enamel substrates, such as composition, density, and microhardness, are similar. Moreover, the bovine and human tooth substrates showed similar behavior regarding staining effects [17, 18].

The white spot lesion is an initial enamel caries caused by alterations in the refractive index of light within the subsurface demineralized enamel [1]. As a result, a whitish appearance is exhibited, which differs in color from the surrounding sound enamel, leading to an undesirable esthetic appearance. The resin infiltration is a minimally invasive treatment to resolve a white spot lesion. A resin-infiltrated agent with a reflective index similar to sound enamel is applied to the lesion. This allows the resin to penetrate and fill the subsurface porosity of the white spot enamel lesion. This method will arrest and transform the whitish and opaque appearance into a smooth and shiny appearance, similar to sound enamel [8, 19].

From the results of this study, immersion in the staining beverage caused significant color changes in the resin-infiltrated specimens, according to the exposure time. All results demonstrated color change more than the perceptibility threshold (ΔE = 1) [20]. The perceptibility threshold of a color difference is visually detectable with little clinical value [21]. Color alterations were evidenced in all groups during their respective immersion periods. Only for the artificial saliva groups with all immersion periods, the color alterations were less than the acceptability threshold (ΔE = 3.3) [20, 22]. On the other hand, the color changes for groups immersed in the staining beverages were higher than the acceptability threshold. The acceptability threshold is a color difference that is an unacceptable alteration to dental esthetics [23]. Therefore, staining beverages might interfere with the dental aesthetic of the teeth.

Regarding the resinous materials’ sorption and solubility properties, the exposure of these materials to dyes and acidic solutions may degrade resin monomers by swelling, plasticization, softening, oxidation, hydrolysis, and affect color stability [24]. Even with the immersion of specimens in the artificial saliva, minor color changes that spectrophotometers could detect could still be found in this study. The color changes (ΔE) were more than the perceptibility threshold. This change might be visually detectable. However, it does not interfere with the dental esthetic because that change is less than the acceptability threshold.

Corresponding with a previous study, they found that wine and coffee immersion led to a notable increase in color change (ΔE) of the demineralized enamel specimens infiltrated with low-viscosity resin [25]. Typically, one of the factors influencing the discoloration of resin-based materials is the chemical properties of their monomers and the presence of fillers. The main composition of the ICON infiltrated agent comprises unfilled TEGDMA. Venz and Dickens conducted a 6-month study on water absorption in polymers with various monomers commonly used in dental materials (TEGDMA, Bis-GMA, UDMA, HMDMA). Their findings indicated that TEGDMA exhibited the highest water absorption rates (TEGDMA > Bis-GMA > UDMA > HMDMA) [26]. This phenomenon was attributed to the hydrophilic ether linkage inherent in TEGDMA [27]. Furthermore, the resin composites with higher proportions of TEGDMA tend to exhibit elevated water absorption levels. The hydrophilic properties of TEGDMA are responsible for its susceptibility to water absorption and environmental staining [28–31]. Another contributing factor is the presence of fillers. The previous study has shown that resin-based materials containing fillers are less susceptible to staining than their filler-free. Consequently, pure resin is more susceptible to discoloration [32–34]. It can be reasonably inferred that the ICON infiltrant is particularly susceptible to staining.

In this study, wine, coffee, Coca-Cola^®^, and green tea were selected as dye-testing substances due to their frequent consumption and recognized strong staining properties. Immersion in all these beverages resulted in significant color alterations, intensifying as the immersion duration increased. A previous study reported that beverages with lower pH values tend to cause more substantial stain penetration compared to those with higher pH values [25]. In the attempt to describe this point, In lab^®^ pH electrodes (Mettler-ToLedo, Greifensee, Switzerland) and pH meters (ORION Star A111 mv/pH METER, BAS, Tokyo, Japan) were used to determine the pH of the solution used in this study. The beverages used in this study exhibited various pH levels ranging from 2.4 to 6.7: Coca-Cola^®^ had the lowest pH (pH = 2.4), followed by wine (pH = 3.6), coffee (pH = 5.4), and green tea Itoen with the highest pH (pH = 6.7). The acidic properties of these beverages could potentially affect the surface integrity of resin-infiltrated enamel, possibly leading to microleakage at the interface and making the infiltrated surfaces more susceptible to staining. Additionally, lower pH levels can soften polymeric materials [35, 36], facilitating pigment adsorption on the resin surface [37]. Therefore, the pronounced discoloration observed in the cola group might be attributed to its lowest pH among all the beverages. On the contrary, the group exposed to green tea, which has the highest pH, exhibited the least amount of discoloration. Moreover, the alcohol’s presence in wine influences its staining potential. The mouth rinses containing alcohol could elevate resin composite absorption and dissolution tendencies, as reported by Almeida GS [38]. The alcohol can roughen and degrade the surface and soften the resin matrix [39, 40], ultimately leading to increased staining [41, 42]. Furthermore, the alteration in color of resin materials could be associated with the assimilation and/or adherence of the coloring agent of the beverages [11]. Tannins are a class of polyphenolic compounds found in beverages like red wine, tea, and coffee. Tannins can bind to proteins on surfaces, and in the case of dental materials, they may contribute to staining. Tannins are known for their high potential to cause discoloration [25, 43]. Therefore, the experimental findings show that immersing specimens in wine results in more pronounced color changes, particularly with longer immersed durations. Tea has a natural tannin, which is a significant staining in resin material after immersion [44, 45]. However, the Wheeler SR’s study reports that the tea extracts do not contain tannin [46]. From the experiment results, green tea, which has a light green color, exhibited no significant color differences in the immersion period from 7 to 28 days. It can be assumed that the green tea solution used in this experiment may not have as high a concentration of the colorant components as other solutions. Coffee was selected as a staining beverage in this experiment because of its regular consumption in daily life. It was associated with color changes in the resin material [47]. The polymer network could interact compatibly with the brown coloring agent [11, 25]. Moreover, enamel staining due to food substances was observed on the surface. Meanwhile, for dentin and cementum, the staining penetrated more. Coffee and soy sauce caused more staining on the calcified dental tissues than cola beverage and tea. Additionally, the duration of staining was directly correlated with the depth of discoloration, as Chan KC’s study [48]. This compatibility promotes the adsorption and penetration of the coloring agent, potentially explaining the observed staining phenomenon in the coffee group.

After 28 days of immersion, this study showed that the color changes could be minimized by polishing with pumice powder. Nevertheless, all specimens submerged in the staining beverage were presented with more color alteration than artificial saliva immersion. However, the main reason for the discoloration from the staining beverage might be prominently from an external stain, except for the coffee group. Consistent with the study of Lee J et al., the resin infiltration-treated specimens showed internal and external staining after four weeks of immersion in four different beverages (coffee, grape juice, iced tea, and distilled water) [49]. It can be assumed that discoloration from beverages is not only confined to the external surfaces of teeth, but it can also penetrate the infiltrated enamel surfaces. The presence of the unpolymerized monomer in the oxygen inhibition zone and the surface treatment of resin infiltration could enhance the permeation and absorption of dye staining from beverages into the specimen [25]. This is reflected in both external and internal color alterations [50]. After the polishing procedure, the outcome in the coffee group indicated a lack of significant reduction in color change. Moreover, the chromogens in the food and beverages significantly influenced the color alterations of surfaces treated with resin infiltration compared to the acidity [51] and alcohol content [25]. The colorant components of coffee are complex and arise from a combination of various compounds. Coffee staining may occur through the adsorption and absorption of coffee dye onto and into the organic phase of the resin material [44]. Adsorption is a surface phenomenon in which atoms, ions, or molecules from a substance adhere to a surface (of the adsorbent). In contrast, absorption is a process that occurs when molecules pass into a bulky material. The particles diffuse or dissolve into another substance. Once dissolved, the molecules cannot be easily separated from the absorbent. The physical and chemical properties of the material surface affect the sorption process. The chemical characteristics, particularly in terms of polarity and ionization of a functional group, suggest that coffee dye might be compatible with the polymer phase within the resin material [47, 52]. Dietschi D et al. suggested that coffee contains yellow color-inducing substances with low polarity, which are released and penetrate into the organic component of the materials [34]. Therefore, after repolishing with pumice in this study, the specimens immersed in coffee continued to exhibit significantly higher staining than other beverages. Considering the limitations of this study, future research should evaluate the effectiveness of daily tooth brushing using toothpaste with different levels of Relative Dentin Abrasivity (RDA) in removing external stains. Nevertheless, dentists should advise the patients about the potential for color alteration of resin infiltration treated white spot lesions caused by the colorant in food and beverages and avoid the consumption to increase the longevity of esthetics outcome.

## Conclusion

The immersion of resin infiltration-treated bovine teeth with artificial white spot lesions in staining beverages (wine, coffee, green tea, and Coca-Cola) induced a notable color alteration after 7 days of immersion compared to artificial saliva. The patterns of color changes in specimens immersed for up to 28 days varied depending on the type of beverage. However, the staining impact was minimized through polishing with pumice powder, except for the coffee group.

## Data Availability

Further information on the data set and materials is available from the corresponding author upon reasonable request.
